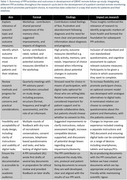# A patient‐centred approach to translate remote cognitive assessments into clinical practice

**DOI:** 10.1002/alz70863_110468

**Published:** 2025-12-23

**Authors:** Jasmine Blane, Shona Forster, Anna Lucas, Sameera Shabir, Grace Gillis, Clare E Mackay

**Affiliations:** ^1^ Oxford Health NHS Foundation Trust, Oxford, Oxfordshire UK; ^2^ University of Oxford, Oxford, Oxfordshire UK; ^3^ BRC Dementia Theme PPI Advisory Group, Oxford, Oxfordshire UK; ^4^ Department of Psychiatry, Oxford Centre for Human Brain Activity (OHBA), Wellcome Centre for Integrative Neuroimaging, University of Oxford, Oxford, Oxfordshire UK; ^5^ Wellcome Centre for Integrative Neuroimaging, University of Oxford, Oxford UK

## Abstract

**Background:**

Despite a proliferation of computerised and remote monitoring assessments offering opportunities to provide valuable data to researchers and clinicians, there are significant concerns about feasibility of such tools within a clinical population. A patient‐centred design is essential to ensure these tools are effective and well tolerated by a memory clinic population, whilst producing high quality data. Our aim was to integrate Patient and Public Involvement (PPI) systematically in the development of a remote monitoring study for patients of the Oxford Brain Health Clinic (OBHC; O'Donoghue et al., 2023).

**Method:**

A comprehensive approach was taken to engage people living with dementia and mild cognitive impairment, their relatives, as well as volunteers interested in dementia research. This was achieved through a workshop and survey to explore the relevance of the research and potential assessments, by user‐testing methods to refine study design, by co‐producing the study aims and documents with a lay member, and through regular consultations with a PPI panel to review progress and practical considerations (Table 1).

**Result:**

PPI activities were instrumental in shaping the research, with two key themes emerging: relevance of outcome measures and choice. The need for clear, relevant, prognostic information was stressed, alongside high‐priority outcome measures such as daily activity, memory, relationships, and care needs. Contributors highlighted the importance of choice in the types of assessments, the means of participation, and to what extent there should be relative involvement. Contributors suggested improvements to digital tasks including increasing device compatibility and the need for analogue alternatives. This resulted in a study design which maximises patient/relative choice while collecting data relating to high‐priority outcome measures. This study design was found by our PPI consultants to be generally acceptable and feasible.

**Conclusion:**

By integrating PPI, we have created a patient‐centred remote monitoring study that is reflective of the needs and preferences of patients and their relatives. Although embedding PPI requires time and resources, it is invaluable for studies involving digital assessments within clinical populations and significantly shaped this research, challenged existing assumptions, and led to more acceptable outcomes, which should ultimately result in greater inclusion.